# Modulatory Effect of Beneficial Enterococci and Their Enterocins on the Blood Phagocytes in Murine Experimental Trichinellosis

**DOI:** 10.3390/life13091930

**Published:** 2023-09-18

**Authors:** Miroslava Vargová, Viera Revajová, Andrea Lauková, Zuzana Hurníková, Emília Dvorožňáková

**Affiliations:** 1 Institute of Parasitology, Slovak Academy of Sciences, 04001 Košice, Slovakia; vargovam@saske.sk (M.V.); hurnikz@saske.sk (Z.H.); 2 Department of Morphological Disciplines, University of Veterinary Medicine and Pharmacy in Košice, 04181 Košice, Slovakia; viera.revajova@uvlf.sk; 3 Institute of Animal Physiology, Centre of Biosciences, Slovak Academy of Sciences, 04001 Košice, Slovakia; laukova@saske.sk

**Keywords:** *Enterococcus*, enterocin M, durancin-like, *Trichinella spiralis*, phagocytosis, respiratory burst

## Abstract

Bacteriocins (enterocins) represent a new therapeutic strategy in various intestinal and non-intestinal infections. In antiparasitic defence, an oxidative inflammation of phagocytes is effective in destroying new-born *Trichinella spiralis* larvae. The strains *Enterococcus faecium* CCM8558 and *E. durans* ED26E/7 and their enterocins, enterocin M and a durancin-like enterocin, respectively, were administered daily, and mice were then infected with *T. spiralis* larvae on the seventh day of treatment. Phagotest and Bursttest kits were used to detect the phagocytosis and respiratory burst in blood leukocytes. *T. spiralis* infection inhibited phagocytosis from day 11 post-infection (dpi) during the migration of new-born larvae into the muscles. *E. faecium* CCM8558, *E. durans* ED26E/7, and the durancin-like enterocin increased phagocytic activity from day 11 dpi. Both strains and their enterocins (enterocin M and durancin-like) stimulated the ingestion capability of phagocytes from 18 to 32 dpi. Enterococci/enterocins therapy prevented a reduction in cells with respiratory burst caused by *T. spiralis* infection from 11 dpi. The enzymatic activity of phagocytes was stimulated on 18 and 25 dpi, particularly by *E. faecium* CCM8558 and enterocin M. Enterocin M and the durancin-like enterocin were as effective in stimulating phagocytosis as the bacterial strains that produce them. The stimulation of phagocytosis could contribute to decreased larval migration and reduced parasite burden in the host.

## 1. Introduction

Probiotic bacteria significantly influence parasite settlement, pathogenicity, and the development of parasitic infection [1]. Probiotic bacteria counteract the development of parasitic infection by strengthening the intestinal barrier and modulating the intestinal microbiota, producing antimicrobials (bacteriocins), modulating the mucosal immune system, and increasing enzymatic activity [2]. Probiotic bacteria modulate both the local and systemic immune response by inhibiting inflammation, stimulating phagocytosis, regulating TLR (Toll-like receptor) expression, activation of antigen-presenting cells, and lymphocyte proliferation. At the same time, they increase the production of specific IgA antibodies in the gastrointestinal tract and are involved in maintaining Th1/Th2 balance [3]. However, the whole bacterial cell may not be required to act immunomodulatory, it might even be replaced by its ribosomally produced antimicrobial proteins—bacteriocins [4]. 

Bacteriocins are membrane-active peptides that damage cell membrane integrity, leading to leakage of intracellular solutes and subsequent cell death. The importance of bacteriocins lies in maintaining homeostasis and also in preventing inflammatory or respiratory diseases, systemic infections, and cancer [5]. Bacteriocins are produced by lactic acid bacteria, so many fermented foods are also their source. The large genus *Enterococcus* is one of the lactic acid bacteria. *Enterococcus* strains produce various bacteriocins (enterocins) that can have potential use as preservatives for food, pharmaceuticals, and nutritional and veterinary drugs [6,7,8]. There are many studies describing the inhibitory activity of bacteriocins from lactic acid bacteria against various viruses, their mechanism of action, and their synergistic and antagonistic effects [9,10,11,12]. The antiparasitic potential of bacteriocins has only been reported in leishmaniosis [13], and in our laboratory, in trichinellosis [14]. The immunomodulatory activity of bacteriocins in parasitic infections has not been studied.

Trichinellosis is one of the most neglected food-borne parasite zoonoses worldwide, which is caused by infectious larvae of *Trichinella* spp. [15]. It is spread in Europe, Southeast Asia, North and South America, New Zealand, and North Africa. Outbreaks have been reported in 55 countries, with an annual global average of 5751 cases and five deaths [16]. The global burden of trichinellosis using disability-adjusted life years was estimated to be 76 per billion persons per year (95% credible interval: 38–129) [17]. Adult stages of *Trichinella* parasitize the small intestine of the host and the new-born larvae (NBL) migrate through the blood and lymphatic circulation into the striated muscles. The severity of the disease depends on the number of infectious doses received, the *Trichinella* species, their location, and the degree of tissue damage. Trichinellosis chemotherapy with common anthelmintics is effective against adult worms in the intestine but not against migrating and muscle larvae. The efficacy of available benzimidazole therapy is insufficient against encapsulated larvae due to low water solubility and anthelmintic resistance, and it is contraindicated for children and pregnant women [18]. It is therefore necessary to develop new methods to control this disease. Therefore, the antiparasitic potential of natural proteins [19], similar to bacteriocins, has been increasingly used in recent years. 

As an alternative biological therapy for trichinellosis, the protective and immunomodulatory effects of enterocins (enterocin M, durancin-like) produced by beneficial *Enterococcus* strains were examined. Enterocins may exert beneficial effects on health through modulation of phagocytosis. Phagocytosis is an essential process for initiating immune responses against infection. Phagocytes play roles in both innate and acquired immunity, they initiate the killing pathway of invaded pathogens by activating oxidase enzymes in phagolysosomes. In the antiparasitic defence in trichinellosis, the oxidative burst of phagocytes (especially hydrogen peroxide) is effective in the destruction of new-born larvae migrating through the blood and lymphatic channels to the striated muscles of the host [20,21]. Granulocytes eliminate NBL and muscle larvae through both oxygen-dependent and antibody-dependent cytotoxic mechanisms [22,23,24]. Therefore, in our experiment, we evaluated the phagocytic and oxidative activity of blood leukocytes after therapy with enterococci/enterocins.

## 2. Materials and Methods

Ethical approval

All animal housing and experiments were conducted in strict accordance with current Slovak ethical rules, the Guidelines for Care and Use of Laboratory Animals of the Institute of Parasitology SAS, and the State Veterinary and Food Administration of the Slovak Republic (No. Ro-1604/19-221/3).

Beneficial enterocin-producing strains and their enterocins

The beneficial strain *Enterococcus faecium* AL41 = CCM 8558 is an environment-derived strain [25] (isolated and characterized at the Centre of Biosciences of the Slovak Academy of Sciences, Institute of Animal Physiology (CBs SAS IAP), Košice, Slovakia and deposited (deposed) in the Czech Culture Collection of Microorganisms, Brno, Czech Republic—CCM 8558). It produces enterocin M (Ent M) with a wide inhibitory spectrum (activity 51,200 AU/mL) and beneficial (probiotic) properties, i.e., stimulates the host’s unspecific immunity. 

The beneficial strain *Enterococcus durans* ED26E/7 [26] was isolated from traditional ewe’s milk lump cheese at the Research Dairy Institute, Žilina; RDI, Žilina, Slovakia, and identified, characterized and prepared for the experiment at CBs SAS IAP, Košice, Slovakia. The strain produces a durancin-like enterocin with a wide antimicrobial activity (25,600 AU/mL).

All strains were checked according to EFSA rules. They were prepared for the experiment as follows: cultivation in MRS broth (Merck, Eppelheim, Germany) at 37 °C for 24 h. After centrifugation of the culture (10,000× *g*/30 min), the supernatant was resuspended to a concentration of 10^9^ colony-forming units per ml (CFU/mL) in Ringer’s solution (Merck, Eppelheim, Germany, pH 7.0). The selective media ME-Enterococcus agar (Difco, Thermo Fisher Scientific, Roskilde, Denmark) and/or MRS agar (Merck, Eppelheim, Germany) were used to check the purity of the strains. The concentration stability of the cultures was verified for 1 week at 4 °C.

Both enterocins (enterocin M and durancin-like), prepared according to Mareková et al. [27], belong to group II.a (pediocin-like enterocins) [28,29], are thermostable with a wide spectrum of inhibitory effects, and retain their activity at low temperatures. They have a protein character and do not leave residues. The protein content of both enterocins was 2 mg/mL.

Experimental design

The experiment was performed on 8-week-old male BALB/c mice (*n* = 126), free of pathogens, weighing 18–20 g (VELAZ, Prague, Czech Republic). The mice were maintained on a commercial diet and a 12 h light/dark regime at room temperature (22–24 °C) and 56% humidity.

**Figure 1:**
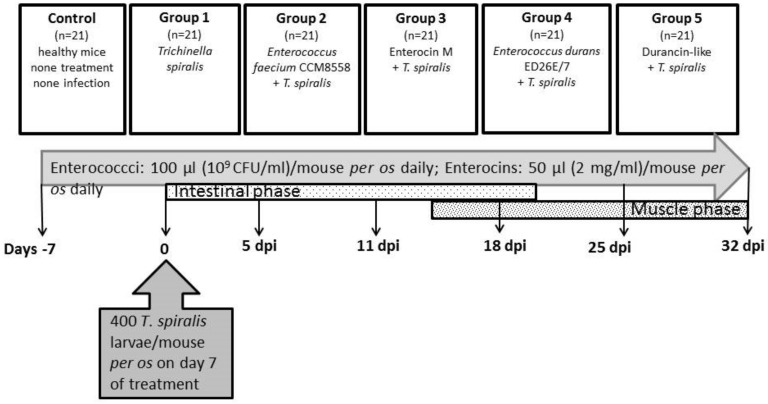
Scheme showing the experiment.

There were six groups of animals in the experiment (Figure 1): Control (*n* = 21)—no treatment and no infection; Group 1 (*n* = 21)—*T. spiralis* infection, no treatment; Group 2 (*n* = 21)—*E. faecium* CCM8558 + *T. spiralis*; Group 3 (*n* = 21)—enterocin M + *T. spiralis*; Group 4 (*n* = 21)—*E. durans* ED26E/7 + *T. spiralis*; Group 5 (*n* = 21)—durancin-like + *T. spiralis. Enterococcus* strains (10^9^ CFU/mL in 100 µL) and enterocins (50 µL) were administered daily per os. Mice were challenged with 400 *T. spiralis* larvae (reference isolate *T. spiralis* ISS 004, maintained at our institute) on the 7th day of treatment. Samples of blood and tissues (the small intestines, muscles) were obtained from mice (3 mice per group) prior to infection: days −7 and 0 and post-infection: 5, 11, 18, 25, and 32 dpi.

Phagocytosis assay

The phagocytic activity of blood monocytes and granulocytes was detected with a Phagotest kit according to the manufacturer’s instructions (Glycotope Biotechnology, Heidelberg, Germany). Briefly [21], 100 µL of heparinized whole blood and 20 µL of FITC-labelled *Escherichia coli* (4 × 10^7^) was incubated at 37 °C/10 min. After stopping the reaction on ice and 100 µL of quenching solution, the cells were washed twice and incubated in 2 mL of lysis solution at RT/20 min. After washing the cells, 200 µL of DNA staining solution was added to the cells for incubation on ice/10 min. Phagocytosis was analysed using flow cytometry (FACScan, Becton Dickinson Biosciences, Heidelberg, Germany). Phagocytic activity was assessed as the percentage of phagocytic cells in the total population (ingested one or more bacteria per cell) and as the phagocytic activity of individual cells (the number of ingested bacteria per cell, measured using fluorescence intensity, GeoMean).

Respiratory burst assay

The oxidative burst activity of blood monocytes and granulocytes was quantified with a Phagoburst kit according to the manufacturer’s instructions (Glycotope Biotechnology, Heidelberg, Germany). Briefly [21], 100 µL of heparinized whole blood was incubated on ice for 10 min, followed by the activation of cells with 20 µL of stimulants (unlabelled opsonized *E. coli* bacteria; protein kinase C ligand phorbol-12-myristate-13-acetate; N-formyl-MetLeuPhe). The negative control was cells without the stimulant. Samples were incubated at 37 °C/10 min, and then 20 µL of substrate dihydrorhodamine was added and incubated at 37 °C/10 min. The reaction was then stopped with 2 mL of lysis solution at RT/20 min. After washing the cells, 200 µL of DNA staining solution was added to the cells followed by incubation on ice for 10 min. The respiratory burst was analysed using flow cytometry (FACScan, Becton Dickinson Biosciences, Heidelberg, Germany). The oxidative activity of phagocytes was assessed as the percentage of phagocytic cells producing reactive oxidants and as their individual enzymatic activity (measured using fluorescence intensity, GeoMean).

Isolation of intestinal worms

Pieces (5–10 cm) of the small intestine were incubated in 0.9% saline on sieves in conical glasses at 37 °C overnight. Then, gut pieces were removed and sedimented worms were counted under a stereomicroscope (60× magnification).

Isolation of muscle larvae

Whole eviscerated carcasses were homogenized and digested with artificial juice (1% pepsin and 1% HCl, both from Sigma-Aldrich, Hamburg, Germany) at 37 °C/4h according to Kapel and Gamble [30]. After sedimentation (20 min), the sediment was filtered (180 µm sieve) and washed with tap water into a conical glass. Larvae present in the sediment were counted in a gridded Petri dish under a stereomicroscope (40× magnification).

Statistical evaluation

One-way ANOVA and Tukey’s post hoc test were performed using Statistica 6.O (Stat Soft, Tulsa, OK, USA). The tests were used in the statistical processing of the results for comparison between two groups at each time point. A value of *p* < 0.05 was considered statistically significant. 

## 3. Results

### 3.1. Phagocytic Activity of Blood Polymorph Nuclear Leukocytes (PMNL)

**Figure 2:**
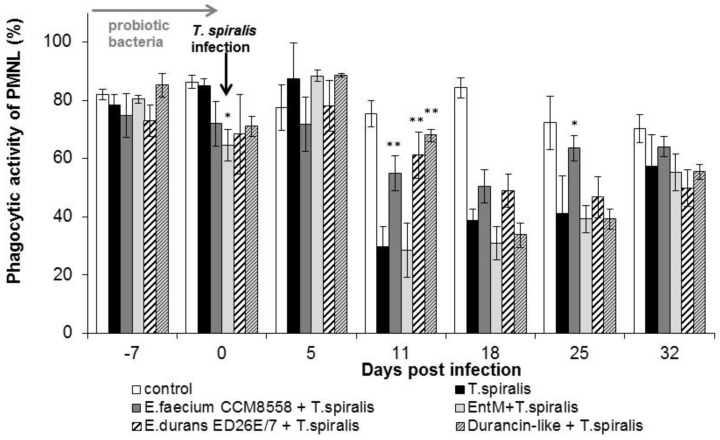
Percentage of PMNL in mice treated with enterococci/enterocins and infected with *T. spiralis*. * *p* < 0.05; ** *p* < 0.01—significantly different compared with *T. spiralis.*

The phagocytic activity of leukocytes (Figure 2) in infected mice without therapy was significantly suppressed from 11 dpi. compared with healthy mice. However, a high percentage of active phagocytes was noted at 11 dpi. in the groups with *E. faecium* CCM8558, *E. durans* ED26E/7, and the durancin-like enterocin, with a significant increase (*p* < 0.01) compared with the *T. spiralis*-infected group without therapy. This stimulatory effect of enterococci lasted until 25 dpi.

**Figure 3:**
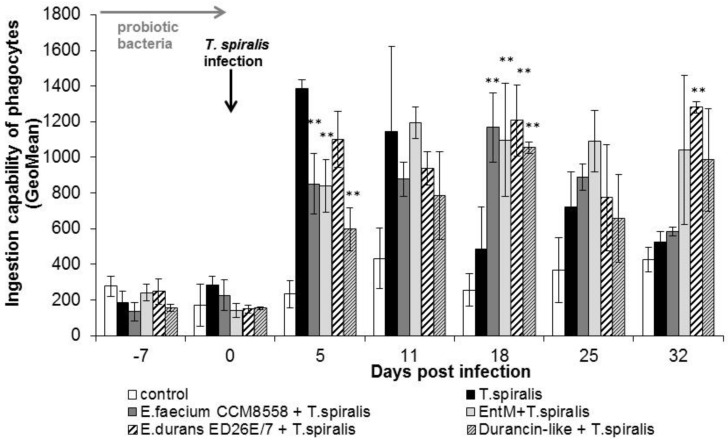
Individual cellular phagocytic activity of PMNL in mice treated with enterococci/enterocins and infected with *T. spiralis*. ** *p* < 0.01—significantly different compared with *T. spiralis.*

The individual ingestion ability of blood leukocytes (Figure 3) in groups of mice infected with *T. spiralis* without therapy or with enterococci/enterocins treatment was significantly increased at the beginning of the intestinal phase (from 5 to 11 dpi.). In the following days, cellular phagocytic ability was inhibited in *T. spiralis*-infected mice without therapy. In the groups with enterococci/enterocins treatment, the values remained significantly (*p* < 0.01) increased from 18 dpi. This stimulatory effect lasted until the end of infection and with maximum after *E. durans* ED26E/7 and enterocin M applications.

### 3.2. Metabolic Activity of Blood Polymorph Nuclear Leukocytes (PMNL)

**Figure 4:**
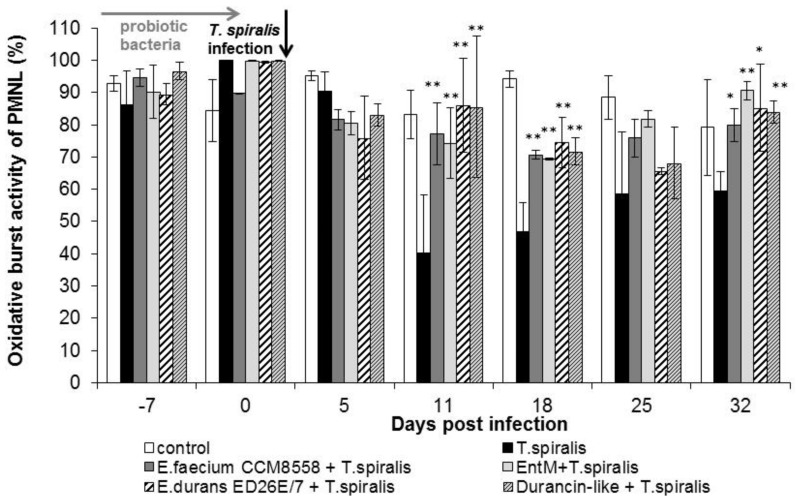
Percentage of phagocyte oxidative burst in PMNL in mice treated with enterococci/enterocins and infected with *T. spiralis*. * *p* < 0.05; ** *p* < 0.01—significantly different compared with *T. spiralis.*

A high percentage of cells with an oxidative burst (Figure 4) were observed even before infection (0 dpi.) in all groups. The percentage of metabolic active cells remained significantly (*p* < 0.01) increased from 11 dpi. in all groups treated with enterococci/enterocins. However, *T. spiralis* infection significantly reduced the percentage of active cells in the intestinal phase of infection (from 11 to 18 dpi), and the lowest values were found in infected mice without therapy until the end of the experiment. Enterocin M and the durancin-like enterocin stimulated the oxidative burst of blood phagocytes as effectively as their producing bacterial strains.

**Figure 5:**
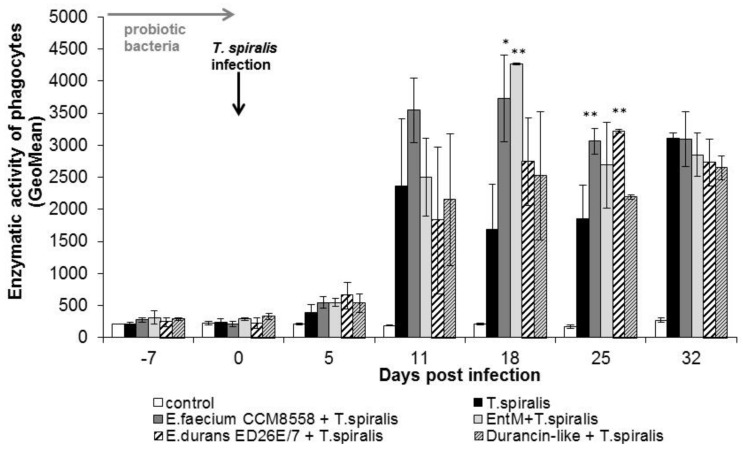
Enzymatic activity of phagocytes in PMNL in mice treated with enterococci/enterocins and infected with *T. spiralis*. * *p* < 0.05; ** *p* < 0.01—significantly different compared with *T. spiralis.*

The individual metabolic activity of leukocytes (Figure 5) was significantly (*p* < 0.01; *p* < 0.05) increased from 11 dpi. in all groups compared with healthy mice. This stimulatory effect persisted until the end of *T. spiralis* infection. Enzyme production reached a maximum at 18 dpi. in mice stimulated with *E. faecium* CCM8558 and enterocin M. At the end of infection, enzymatic activity was equally increased in infected-untreated and infected-treated mice.

### 3.3. Development of Parasitic Infection during the Intestinal and Muscular Phase of Trichinellosis

The number of adult worms during the intestinal phase of trichinellosis was lower in mice with enterococci/enterocins treatment compared with the infected mice without therapy (Table 1). The number of adults in the intestine of mice reached maximum values in all groups at 5 dpi.; however, a significant (*p* < 0.01; *p* < 0.05) reduction was observed in mice treated with enterocin M, *E. durans* ED26E/7, and the durancin-like enterocin. The greatest reduction in the number of adults in the small intestine of mice occurred at 11 dpi. (*p* < 0.01) in mice with *E. faecium* CCM8558 application (54.5%). Its enterocin M (46%) also had a similar effect. *E. durans* ED26E/7 achieved a 35.7% antiparasitic effect, while its durancin-like enterocin showed only 16.4%.

At the beginning of the muscle phase, only low numbers of larvae were obtained at 18 dpi. The maximum counts were detected at the end of the experiment at 32 dpi. (Table 1). The significantly greatest reduction in the number of larvae was found at 25 dpi. (*p* < 0.01), namely, in the groups with *E. faecium* CCM8558 (66.5%) and enterocin M (51.5%). The anti-larval effect of enterococci/enterocins therapy lasted until 32 dpi. 

## 4. Discussion

Intestinal microbiota significantly affects the completion of the parasite life cycle in the gut, influencing the development of larvae into adult individuals and their reproductive capacity [1]. Probiotic organisms are able to modulate their physical–chemical environment, pH, intestinal motility, and mucus secretion [31,32], which are major components of intestinal physiology in host defence against worms [33]. The beneficial activity of a probiotic organism depends on the metabolites it produces. They secret various enzymes (β-galactosidase, proteases, lipases) and bacteriocins with antimicrobial activity. The production of bacteriocins and hydrogen peroxide [31,34] can prevent parasites from entering the intestinal epithelial cells, i.e., the site for the moulting of Trichinella larvae, transformation into adults, and reproduction [35]. 

During the intestinal phase of trichinellosis in mice treated with enterococci/enterocins, we observed a significant reduction in adult worms compared with infected mice without therapy. In the early phase of trichinellosis (at 5 dpi), when adult worms are established in the epithelium of the small intestine and females give birth to live larvae in high numbers (500–1500 NBL/female; [36]), the enterocins themselves are also effective in expelling worms. Enterocin M reduced the number of adults by 43.8%, and the durancin-like enterocin reduced the number of adults by 32.5%. Enterocin M retained its effectiveness even in the developed intestinal phase on the 11th dpi. (46%), but the durancin-like enterocin inhibited the presence of worms by only 16.4%. The strain *E. durans* ED26E/7 showed a balanced antiparasitic effect on adults (34.1 and 35.7%) during the intestinal phase, but *E. faecium* CCM8558 maximally stimulated worm expulsion only in the developed intestinal phase at 11 dpi. (54.5%). A similar trend indicating the reduction in Trichinella in the intestine after the application of *E. faecium* CCM8558 (53%) and *E. durans* ED26E/7 (38%) was also noted [37]. Oral application of other lactic acid strains *Lactobacillus casei* ATCC 7469, *L. acidophilus* P110, and *L. plantarum* P164 reduced trichinella in the intestine of mice by 36–58% [38,39]. *Lactobacillus casei* ATCC 393 and *L. paracasei* CNCM caused a significant reduction in adults in the gut [40]. However, the application of *Enterococcus faecalis* CECT7121 did not affect adult *T. spiralis* burden in the gut in mice [41].

In the muscular phase of trichinellosis, we found the greatest reduction in the number of larvae at 25 dpi. in mice treated with *E. faecium* CCM8558 (66.5%), enterocin M (51.5%), *E. durans* ED26E/7 (42.4%), and the durancin-like enterocin (31.6%). The anti-larval effect of enterococci/enterocins therapy lasted until 32 dpi. (*E. faecium* CCM8558—55.7%, enterocin M—39.6%, *E. durans* ED26E/7—36.3%), with the exception of the durancin-like enterocin (15%). These results confirmed the significant antiparasitic effect of enterocin M alone, which was comparable to its producer *E. faecium* CCM8558. A similar inhibitory effect of enterococci on *Trichinella* muscle larvae was also evaluated, which showed a reduction of 65% for *E. faecium* CCM8558 and 50% for *E. durans* ED26E/7 [37]. Some strains of lactobacilli (*L. casei* ATCC7469, *L. casei* Shirota, and *L. plantarum* P164) also had an anti-larval effect in trichinellosis [38,39,42], but we cannot generalize these results because the beneficial effects of probiotic bacteria are strain-specific [43].

We hypothesize that in our work, enterococci/enterocins therapy inhibited NBL motility and migration. Lactic acid bacteria, including enterococci, form lactic acid, acetic acid, protein enterocins, and hydrogen peroxide, which are necessary for the destruction of pathogens [44] and are also used in the antiparasitic defence of the host [45,46], as documented by a significant reduction in larval burden after enterococci/enterocin therapy. It is probable that enterocins disrupt the cell membrane not only of the target cells [47] but also the surface structures of larvae. Enterocin M and the durancin-like enterocin could act through permeabilization changes in the cuticle layers of the larvae, the formation of pores that allow the influx of ions, oxidative damage to internal structures, and the bioenergetic collapse of the organism.

A very important aspect of the mechanism of action of probiotic bacteria is their immunomodulating abilities. From an immunological aspect, bacteriocins represent a new strategy for suppressing or preventing various intestinal and extra-intestinal infections. The host initiates an immune response to the parasite at the intestinal level, where the parasite and intestinal microbiota interact, and the host’s immune system changes as a result of both. Phagocytosis by blood leukocytes is the host’s defence basic tool against pathogens including parasites [48]. Phagocytes are the central cells in the inflammatory reaction, and their immunostimulation can modulate the course of the inflammatory reaction and subsequent pathological changes [49]. Many studies have investigated the modulatory effect of probiotic bacteria on phagocytosis in recent years. Many strains stimulate the activity of blood phagocytes (monocytes, polymorph nuclear cells), which participate in a mucosal barrier of the intestinal walls, thus inhibiting the transfer of antigens through the intestinal mucosa and supporting the growth of probiotic microbiota [50]. 

Increased phagocytic activity after stimulation with the bacterium *Lactobacillus johnsoni* La1 was recorded four weeks after the disappearance of the probiotic strain from faeces [51]. Similarly, an increase in the phagocytic activity of leukocytes in dogs was found until the 5th week after ending the application of *L. fermentum* CCM7421 and *Bifidobacterium animalis* B/12 [52,53]. Increased phagocytic activity of peripheral blood mononuclear cells (PBMCs) was detected after the application of *Enterococcus faecium* CCM8558 and *Bifidobacterium lactis* HN019 [21,54]. Increased phagocytosis by heterophiles was noted in the study [55], in which chickens infected with *Salmonella enterica* serovar Enteritidis were administered the probiotic strain *Enterococcus faecium* EF55. *Bifidobacterium adolescentis* BB-2 and *Bifidobacterium longum* B-3 strains also confirmed the immunostimulatory effect on the phagocytic activity of macrophages [56]. Granulocytes from the liver and peripheral blood of piglets fed probiotics BIOTHREEPlus (BT) also showed higher phagocytic activity than the control [57]. Consumption of *Bifidobacterium lactis* HN019 significantly stimulated PBMC phagocytosis of NK cells in humans [58]. Sublancin (a glycosylated antimicrobial peptide) produced by *Bacillus subtilis* 168 enhanced the phagocytic activity of peritoneal macrophages in immunosuppressed mice [59].

In our study, parasitic infection with *T. spiralis* during the first week of infection did not change the percentage of phagocytic cells but significantly increased the individual ingestion capacity of phagocytes up to 11 dpi. However, on this day, the phagocytic activity significantly decreased, and a reduction was noted in the population of blood phagocytes in untreated-infected mice until the end of the experiment. Application of enterococci (*E. faecium* CCM8558, *E. durans* ED26E/7) and the durancin-like enterocin increased the number of phagocytes at 11 dpi. and both enterococcal strains and enterocins (enterocin M and durancin-like) stimulated the ingestion capacity of phagocytes during the migratory and muscular phases of infection (from 18 to 32 dpi). A similar stimulatory effect of enterococci on blood phagocytes was noted in another study [21]. 

In our work, the phagocytic activity of leukocytes was most significantly stimulated with the strain *E. faecium* CCM8558 from the 11th dpi., i.e., from the time when the larvae begin their migration until the time of settling in the muscles (25–32 dpi.). While studying immunity genes in the “nurse cell” system around muscle larvae, it was found that encysted larvae are constantly actively connected to the host’s immune response and can induce phagocytosis and oxidative stress [60]. In other studies, the strain *E. faecium* CCM8558 increased the phagocytosis of blood PMNL even after successful colonization of the intestine in horses, rabbits, and laying hens [25,26,61]. Other authors documented the activation of phagocytosis by probiotic strains, mainly lactobacilli, in humans, pigs, and dogs [52,62,63]. A stimulation of phagocytic activity and oxidative burst of neutrophils were recorded during the long-term administration of milk with *L. helveticus* NCDC292, *L. acidophilus* NCDC15, and *L. paracasei* [64]. Not only whole cells but also exopolysaccharides (EPS) from the *Lactobacillus kiferi* strain increased macrophage phagocytic activity and NO production [65]. This study, together with our results after the application of enterocins, point to the fact that bacterial products (EPS, bacteriocins) are able to successfully modulate the immune system.

The oxidative activity of phagocytes, i.e., production of reactive oxidative metabolites, was significantly suppressed in *T. spiralis*-infected and untreated mice at 11–18 dpi. during this period of intensive migration of NBL, which release suppressor substances to ensure successful localization in the host’s muscles. The presence of phagocytosis inhibitory factors was confirmed in the sera of patients with trichinellosis [66]. Enterococci/enterocins therapy in our experiment significantly stimulated the oxidative metabolism of phagocytes precisely during this period of NBL migration from 11 dpi to the end of the experiment, while enzyme production was maximal at 18 dpi. in mice with the application of *E. faecium* CCM8558 and enterocin M. Stimulation of the oxidative metabolism of phagocytes could thus contribute to the destruction of NBL and the reduction in muscle larvae that was recorded on 25 dpi. Neutrophils and macrophages produce nitric oxide (NO), which kills *T. spiralis* larvae. However, during their development, larvae are not equally sensitive to this deadly mechanism [67]. NBL and muscle larvae are easily destroyed within 11 days, but mature muscle larvae (more than 14 days) are resistant to nitric oxide. The first larvae can colonize muscles between 4 and 14 dpi., and subsequently, the proportion of larvae susceptible to NO decreases between 14 and 28 dpi. That means that we can influence the reduction in the parasitic load in the muscles until the 28th dpi. In our work, therapy with enterococci/enterocins stimulated oxidative metabolism in phagocytes from 11 to 18 dpi., which may be associated with the NO effect on larvae resulting in reduced parasitic load.

## 5. Conclusions

A protective effect of enterocins (enterocin M and durancin-like), produced by beneficial *Enterococcus* strains (*E. faecium* CCM8558 and *E. durans* ED26E/7), was examined as an alternative biological therapy for trichinellosis. Enterocinogenic activity was evaluated in the interactions between enterocin application, induction of immune effectors, and elimination of the parasite. Enterocins/enterococci therapy prevented the suppression of phagocytosis of blood PBM during caused by *T. spiralis* infection in the intestinal phase of trichinellosis and stimulated phagocytic activity and oxidative burst during the migration of NBL into muscles. The immunomodulatory effects of enterocin M and the durancin-like enterocin were comparable to their producing strains. 

Knowledge of the immunological modulations induced by beneficial enterocins/enterococci on *T. spiralis* infection is the basis for setting an effective pharmacological strategy that can reduce the risk of parasite infection or supplement classical antiparasitic treatment with a reduced dose of anthelmintics.

## Figures and Tables

**Table 1:** Parasite burden in mice.

Intestinal Phase (Numbers of Adults Per Animal)						
**Days Post-Infection**	**5**		**11**		**18**	
	**(Mean ± S.D.)**	**Reduction (%)**	**(Mean ± S.D.)**	**Reduction (%)**	**(Mean ± S.D.)**	**Reduction (%)**
***T.spiralis***	308 ± 25		213 ± 17		1 ± 3	
***E.faecium*** **CCM8558 + *T.spiralis***	276 ± 9	**10.4**	** 97 ± 18	**54.5**	0 ± 1	100
**Enterocin M + *T.spiralis***	** 173 ± 22	**43.8**	** 115 ± 7	**46**	1 ± 4	0
***E.durans*** **ED26E/7 + *T.spiralis***	* 203 ± 40	**34.1**	** 137 ± 13	**35.7**	0 ± 1	100
**Durancin-like + *T.spiralis***	** 208 ± 22	**32.5**	** 178 ± 13	**16.4**	5 ± 4	0
**Muscle Phase (Numbers of Larvae Per Animal)**						
**Days post-** **Infection**	**18**		**25**		**32**	
	**(Mean ± S.D.)**	**Reduction (%)**	**(Mean ± S.D.)**	**Reduction (%)**	**(Mean ± S.D.)**	**Reduction (%)**
***T.spiralis***	9 ± 2		43,305 ± 1972		50,515 ± 1540	
***E.faecium*** **CCM8558 + *T.spiralis***	42 ± 4	0	** 14,495 ± 1839	**66.5**	** 22,363 ± 1005	**55.7**
**Enterocin M + *T.spiralis***	43 ± 7	0	** 13,013 ± 1598	**51.5**	** 30,513 ± 2468	**39.6**
***E.durans*** **ED26E/7 + *T.spiralis***	30 ± 3	0	** 24,965 ± 3690	**42.4**	** 32,163 ± 2178	**36.3**
**Durancin-like + *T.spiralis***	28 ± 4	0	** 29,636 ± 2330	**31.6**	42,930 ± 2281	**15.1**

* *p* < 0.05; ** *p* < 0.01—significantly different compared with the *T. spiralis*-infected group without treatment.

## Data Availability

The data presented in this study are available within the article.

## References

[1] Berrilli F., Di Cave D., Cavallero S., D’Amelio S. (2012). Interactions between parasites and microbial communities in the human gut. Front. Cell. Infect. Microbiol..

[2] Bajagai Y.S., Klieve A.V., Dart P.J., Bryden W.L. (2016). Probiotics in Animal Nutrition: Production, Impact and Regulation.

[3] Tsai Y.T., Cheng P.C., Pan T.M. (2012). The immunomodulatory effects of lactic acid bacteria for improving immune functions and benefits. Appl. Microbiol. Biotechnol..

[4] Llewellyn A., Foey A. (2017). Probiotic modulation of innate cell pathogen sensing and signaling events. Nutrients.

[5] Bharti V., Mehta A., Singh S., Jain N., Ahirwal L., Mehta S. (2015). Bacteriocin: A novel approach for preservation of food. Int. J. Pharm. Pharm. Sci..

[6] Wu Y., Pang X., Wu Y., Liu X., Zhang X. (2022). Enterocins: Classification, synthesis, antibacterial mechanisms and food applications. Molecules.

[7] Sánchez J., Borrero J., Gómez-Sala B., Basanta A., Herranz C., Cintas L.M., Hernández P.E. (2008). Cloning and heterologous production of Hiracin JM79, a Sec-dependent bacteriocin produced by *Enterococcus hirae* DCH5, in lactic acid bacteria and *Pichia pastoris*. Appl. Environ. Microbiol..

[8] Birri D.J., Brede D.A., Forberg T., Holo H., Nes I.F. (2010). Molecular and genetic characterization of a novel bacteriocin locus in *Enterococcus avium* isolates from infants. Appl. Environ. Microbiol..

[9] Wachsman M.B., Castilla V., de Ruiz Holgado A.P., de Torres R.A., Sesma F., Coto C.E. (2003). Enterocin CRL35 inhibits late stages of HSV-1 and HSV-2 replication in vitro. Antivir. Res..

[10] Todorov S.D., Dicks L.M. (2005). Optimization of bacteriocin ST311LD production by *Enterococcus faecium* ST311LD, isolated from spoiled black olives. J. Microbiol..

[11] Férir G., Petrova M.I., Andrei G., Huskens D., Hoorelbeke B., Snoeck R., Vanderleyden J., Balzarini J., Bartoschek S., Brönstrup M. (2013). The lantibiotic peptide labyrinthopeptin A1 demonstrates broad anti-HIV and anti-HSV activity with potential for microbicidal applications. PLoS ONE.

[12] Al Kassaa I., Hober D., Hamze M., Chihib N.E., Drider D. (2014). Antiviral potential of lactic acid bacteria and their bacteriocins. Probiotics Antimicrob. Proteins.

[13] Abengózar M.Á., Cebrián R., Saugar J.M., Gárate T., Valdivia E., Martínez-Bueno M., Maqueda M., Rivas L. (2017). Enterocin AS-48 as evidence for the use of bacteriocins as new leishmanicidal agents. Antimicrob. Agents Chemother..

[14] Vargová M., Dvorožňáková E., Hurníková Z., Lauková A., Revajová V. (2019). Antiparasitic potential of enterocins and enterocin-producing strains for *Trichinella spiralis* infection. Slov. Veterinársky Časopis.

[15] Bouwknegt M., Devleesschauwer B., Graham H., Robertson L.J., van der Giessen J.W. (2018). Euro-FBP workshop participants. Prioritisation of food-borne parasites in Europe, 2016. Eurosurveillance.

[16] Rostami A., Gamble H.R., Dupouy-Camet J., Khazan H., Bruschi F. (2017). Meat sources of infection for outbreaks of human trichinellosis. Food Microbiol..

[17] Devleesschauwer B., Praet N., Speybroeck N., Torgerson P.R., Haagsma J.A., De Smet K., Murrell K.D., Pozio E., Dorny P. (2015). The low global burden of trichinellosis: Evidence and implications. Int. J. Parasitol..

[18] Yadav A.K., Temjenmongla (2012). Efficacy of *Lasia spinosa* leaf extract in treating mice infected with *Trichinella spiralis*. Parasitol. Res..

[19] Othman A.A., Shoheib Z.S. (2016). Detrimental effects of geldanamycin on adults and larvae of *Trichinella spiralis*. Helminthologia.

[20] Bass D.A., Szejda P. (1979). Mechanisms of killing of newborn larvae of *Trichinella spiralis* by neutrophils and eosinophils: Killing by generators of hydrogen peroxide In Vitro. J. Clin. Investig..

[21] Dvorožňáková E., Bucková B., Hurníková Z., Revajová V., Lauková A. (2016). Effect of probiotic bacteria on phagocytosis and respiratory burst activity of blood polymorphonuclear leukocytes (PMNL) in mice infected with *Trichinella spiralis*. Vet. Parasitol..

[22] Gurish M.F., Humbles A., Tao H., Finkelstein S., Boyce J.A., Gerard C., Friend D.S., Austen K.F. (2002). CCR3 is required for tissue eosinophilia and larval cytotoxicity after infection with *Trichinella spiralis*. J. Immunol..

[23] Beiting D.P., Bliss S.K., Schlafer D.H., Roberts V.L., Appleton J.A. (2004). Interleukin-10 limits local and body cavity inflammation during infection with muscle-stage *Trichinella spiralis*. Infect. Immun..

[24] Bruschi F., Korenaga M., Watanabe N. (2008). Eosinophils and *Trichinella infection*: Toxic for the parasite and the host?. Trends Parasitol..

[25] Lauková A., Kandričáková A., Ščerbová J. (2015). Use of bacteriocin-producing, probiotic strain *Enterococcus faecium* AL41 to control intestinal microbiota in farm ostriches. Lett. Appl. Microbiol..

[26] Lauková A., Chrastinová Ľ., Kandričáková A., Ščerbová J., Plachá I., Simonová M.P., Čobanová K., Formelová Z., Ondruška Ľ., Strompfová V. (2015). Bacteriocin substance durancin-like Ed 26E/7 and its experimental use in broiler rabbits. Maso.

[27] Mareková M., Lauková A., Skaugen M., Nes I. (2007). Isolation and characterization of a new bacteriocin, termed enterocin M, produced by environmental isolate *Enterococcus faecium* AL41. J. Ind. Microbiol. Biotechnol..

[28] Franz C.M., van Belkum M.J., Holzapfel W.H., Abriouel H., Gálvez A. (2007). Diversity of enterococcal bacteriocins and their grouping in a new classification scheme. FEMS Microbiol. Rev..

[29] Ness I.F., Diep D.B., Ike Y., Gilmore M.S., Clewell D.B., Ike Y., Shankar N. (2014). Enterococcal Bacteriocins and Antimicrobial Proteins that Contribute to Niche Control. Enterococci: From Commensals to Leading Causes of Drug Resistant Infection.

[30] Kapel C.M., Gamble H.R. (2000). Infectivity, persistence, and antibody response to domestic and sylvatic *Trichinella* spp. in experimentally infected pigs. Int. J. Parasitol..

[31] Gupta V., Garg R. (2009). Probiotics. Indian J. Med. Microbiol..

[32] Travers M.A., Florent I., Kohl L. (2011). Grellier, Probiotics for the control of parasites: An overview. J. Parasitol. Res..

[33] Khan W.I. (2008). Physiological changes in the gastrointestinal tract and host protective immunity: Learning from the mouse-*Trichinella spiralis* model. Parasitology.

[34] Hertzberger R., Arents J., Dekker H.L., Pridmore R.D., Gysler C., Kleerebezem M., de Mattos M.J. (2014). H_2_O_2_ production in species of the *Lactobacillus acidophilus* group: A central role for a novel NADH-dependent flavin reductase. Appl. Environ. Microbiol..

[35] Gagliardo L.F., McVay C.S., Appleton J.A. (2002). Molting, ecdysis, and reproduction of *Trichinella spiralis* are supported in vitro by intestinal epithelial cells. Infect. Immun..

[36] Pozio E., La Rosa G., Murrell K.D., Lichtenfels J.R. (1992). Taxonomic revision of the genus *Trichinella*. J. Parasitol..

[37] Bucková B., Hurníková Z., Lauková A., Revajová V., Dvorožňáková E. (2018). The anti-parasitic effect of probiotic bacteria via limiting the fecundity of *Trichinella spiralis* female adults. Helminthologia.

[38] Bautista-Garfias C.R., Ixta-Rodríguez O., Martínez-Gómez F., López M.G., Aguilar-Figueroa B.R. (2001). Effect of viable or dead *Lactobacillus casei* organisms administered orally to mice on resistance against *Trichinella spiralis* infection. Parasite.

[39] El-Temsahy M.M., Ibrahim I.R., Mossallam S.F., Mahrous H., Bary A.A., Salam S.A.A. (2015). Evaluation of newly isolated probiotics in the protection against experimental intestinal trichinellosis. Vet. Parasitol..

[40] Boros Z., Băieș M.H., Vodnar D.C., Gherman C.M., Borșan S.D., Cozma-Petruț A., Lefkaditis M., Györke A., Cozma V. (2022). Antiparasitic Action of *Lactobacillus casei* ATCC 393 and *Lactobacillus paracasei* CNCM Strains in CD-1 Mice Experimentally Infected with *Trichinella britovi*. Pathogens.

[41] Schofs L., Sparo M.D., de Yaniz M.G., Lissarrague S., Domínguez M.P., Álvarez L.I., Bruni S.F.S. (2022). Antinematodic effect of *Enterococcus faecalis* CECT7121 using *Trichinella spiralis* as a model of nematode infection in mice. Exp. Parasitol..

[42] Martínez-Gómez F., Fuentes-Castro B.E., Bautista-Garfias C.R. (2011). The intraperitoneal inoculation of *Lactobacillus casei* in mice induces total protection against *Trichinella spiralis* infection at low challenge doses. Parasitol. Res..

[43] Butel M.J. (2014). Probiotics, gut microbiota and health. Med. Mal. Infect..

[44] Lauková A., Chrastinová L., Simonová M.P., Strompfová V., Plachá I., Čobanová K., Formelová Z., Chrenková M., Ondruška L. (2012). *Enterococcus faecium* AL 41: Its Enterocin M and their beneficial use in rabbits husbandry. Probiotics Antimicrob. Proteins.

[45] El-Temsahy M.M. (2001). The effect of changes in the gastric pH value on experimental trichinosis. J. Egypt. Soc. Parasitol..

[46] Jin X., Liu Y., Vallee I., Karadjian G., Liu M., Liu X. (2022). Lentinan-triggered butyrate-producing bacteria drive the expulsion of the intestinal helminth *Trichinella spiralis* in mice. Front. Immunol..

[47] Van Staden A.D., Brand A.M., Dicks L.M. (2012). Nisin F-loaded brushite bone cement prevented the growth of *Staphylococcus aureus* in vivo. J. Appl. Microbiol..

[48] Hübel K., Dale D.C., Liles W.C. (2002). Therapeutic use of cytokines to modulate phagocyte function for the treatment of infectious diseases: Current status of granulocyte colony-stimulating factor, granulocyte-macrophage colony-stimulating factor, macrophage colony-stimulating factor, and interferon-gamma. J. Infect. Dis..

[49] Rosales C., Uribe-Querol E. (2017). Phagocytosis: A fundamental process in immunity. Biomed. Res. Int..

[50] Fedorak R.N., Madsen K.L. (2004). Probiotics and prebiotics in gastrointestinal disorders. Curr. Opin. Gastroenterol..

[51] Donnet-Hughes A., Rochat F., Serrant P., Aeschlimann J.M., Schiffrin E.J. (1999). Modulation of nonspecific mechanisms of defense by lactic acid bacteria: Effective dose. J. Dairy Sci..

[52] Strompfová V., Lauková A., Gancarčíková S. (2012). Effectivity of freeze-dried form of *Lactobacillus fermentum* AD1-CCM7421 in dogs. Folia Microbiol..

[53] Strompfová V., Simonová M.P., Gancarčíková S., Mudroňová D., Farbáková J., Mad’ari A., Lauková A. (2014). Effect of *Bifidobacterium animalis* B/12 administration in healthy dogs. Anaerobe.

[54] Arunachalam K., Gill H.S., Chandra R.K. (2000). Enhancement of natural immune function by dietary consumption of *Bifidobacterium lactis* (HN019). Eur. J. Clin. Nutr..

[55] Levkut M., Revajová V., Lauková A., Ševčíková Z., Spišáková V., Faixová Z., Levkutová M., Strompfová V., Pistl J., Levkut M. (2012). Leukocytic responses and intestinal mucin dynamics of broilers protected with *Enterococcus faecium* EF55 and challenged with *Salmonella* enteritidis. Res. Vet. Sci..

[56] Fan J., Hou Y., Zhou S., Cai X. (2015). Effect of Bifidobacterium on the immunity in BALB/c mice. Wei Sheng Wu Xue Bao.

[57] Azizi A.F.N., Uemura R., Omori M., Sueyoshi M., Yasuda M. (2022). Effects of probiotics on growth and immunity of piglets. Animals.

[58] Chiang B.L., Sheih Y.H., Wang L.H., Liao C.K., Gill H.S. (2000). Enhancing immunity by dietary consumption of a probiotic lactic acid bacterium (*Bifidobacterium lactis* HN019): Optimization and definition of cellular immune responses. Eur. J. Clin. Nutr..

[59] Wang S., Huang S., Ye Q., Zeng X., Yu H., Qi D., Qiao S. (2018). Prevention of cyclophosphamide-induced immunosuppression in mice with the antimicrobial peptide Sublancin. J. Immunol. Res..

[60] Dabrowska M. (2013). Inflammatory phenotype of the nurse cell harboring *Trichinella* spp. Vet. Parasitol..

[61] Lauková A., Styková E., Kubašová I., Strompfová V., Gancarčíková S., Plachá I., Miltko R., Belzecki G., Valocký I., Simonová M.P. (2020). Enterocin M-producing *Enterococcus faecium* CCM 8558 demonstrating probiotic properties in horses. Probiotics Antimicrob. Proteins.

[62] Rask C., Adlerberth I., Berggren A., Ahrén I.L., Wold A.E. (2013). Differential effect on cell-mediated immunity in human volunteers after intake of different lactobacilli. Clin. Exp. Immunol..

[63] Chytilová M., Mudroňová D., Nemcová R., Gancarčíková S., Buleca V., Koščová J., Tkáčiková L. (2013). Anti-inflammatory and immunoregulatory effects of flax-seed oil and *Lactobacillus plantarum*—Biocenol™ LP96 in gnotobiotic pigs challenged with enterotoxigenic *Escherichia coli*. Res. Vet. Sci..

[64] Kapila R., Sebastian R., Varma D.V.P., Sharma R., Kapasiya M., Salingati V., Kapila S., Dang A.K. (2013). Comparison of innate immune activation after prolonged feeding of milk fermented with three species of Lactobacilli. Microbiol. Immunol..

[65] Xiu L., Sheng S., Hu Z., Liu Y., Li J., Zhang H., Liang Y., Du R., Wang X. (2020). Exopolysaccharides from *Lactobacillus kiferi* as adjuvant enhanced the immuno-protective against *Staphylococcus aureus* infection. Int. J. Biol. Macromol..

[66] Bruschi F., Carulli G., Azzarà A., Minnucci S. (1995). Inhibition of neutrophil oxidative metabolism by trichinellosis patient sera. Parasite origin or host induction?. Parasite Immunol..

[67] Gebreselassie N.G., Moorhead A.R., Fabre V., Gagliardo L.F., Lee N.A., Lee J.J., Appleton J.A. (2012). Eosinophils preserve parasitic nematode larvae by regulating local immunity. J. Immunol..

